# Socioeconomic inequality in child health outcomes in India: analyzing trends between 1993 and 2021

**DOI:** 10.1186/s12939-024-02218-z

**Published:** 2024-07-31

**Authors:** Anoop Jain, Rockli Kim, Soumya Swaminathan, SV Subramanian

**Affiliations:** 1 Department of Environmental Health, Boston University School of Public Health, 715 Albany St, Boston, MA 02118 USA; 2 Division of Health Policy & Management, College of Health Science, Korea University, 145 Anam-ro, Seongbuk-gu, Seoul, 02841 South Korea; 3 MS Swaminathan Research Foundation, 3rd Cross Street, Institutional Area, Taramani, Chennai, 600 113 India; 4 Harvard Center for Population and Development Studies, 9 Bow Street, Cambridge, MA 02138 USA; 5 Department of Social and Behavioral Sciences, Harvard T.H. Chan School of Public Health, 677 Huntington Avenue, Boston, MA 02115 USA

**Keywords:** India, Child health, Child diarrhea, Anthropometric failure, National Family Health Survey, Socioeconomic status

## Abstract

**Background:**

The health of India’s children has improved over the past thirty years. Rates of morbidity and anthropometric failure have decreased. What remains unknown, however, is how those patterns have changed when examined by socioeconomic status. We examine changes in 11 indicators of child health by household wealth and maternal education between 1993 and 2021 to fill this critical gap in knowledge. Doing so could lead to policies that better target the most vulnerable children.

**Methods:**

We used data from five rounds of India’s National Family Health Survey conducted in 1993, 1999, 2006, 2016, and 2021 for this repeated cross-sectional analysis. We studied mother-reported cases of acute respiratory illness and diarrhea, hemoglobin measurements for anemia, and height and weight measurements for anthropometric failure. We examined how the prevalence rates of each outcome changed between 1993 and 2021 by household wealth and maternal education. We repeated this analysis for urban and rural communities.

**Results:**

The socioeconomic gradient in 11 indicators of child health flattened between 1993 and 2021. This was in large part due to large reductions in the prevalence among children in the lowest socioeconomic groups. For most outcomes, the largest reductions occurred before 2016. Yet as of 2021, except for mild anemia, outcome prevalence remained the highest among children in the lowest socioeconomic groups. Furthermore, we show that increases in the prevalence of stunting and wasting between 2016 and 2021 are largely driven by increases in the severe forms of these outcomes among children in the highest socioeconomic groups. This finding underscores the importance of examining child health outcomes by severity.

**Conclusions:**

Despite substantial reductions in the socioeconomic gradient in 11 indicators of child health between 1993 and 2021, outcome prevalence remained the highest among children in the lowest socioeconomic groups in most cases. Thus, our findings emphasize the need for a continued focus on India’s most vulnerable children.

**Supplementary Information:**

The online version contains supplementary material available at 10.1186/s12939-024-02218-z.

## Introduction

Indicators of child health have improved throughout India over the past three decades. For example, the burden of child malnutrition – as measured by anthropometric failure and anemia – fell between 1990 and 2017 [1]. The burden of infectious diseases among children has also fallen throughout India over the past few decades. In fact, the number of under-five deaths attributable to diarrhea fell by 86% between 1980 and 2015, a clear indication that diarrhea is being better prevented and treated [2]. 

What remains unknown, however, is the extent to which child health outcomes have changed when examined through the prism of socioeconomic status (SES). A household’s SES is an important predictor of child health [3]. Two of the most commonly used indicators of SES when examining child health are household wealth and maternal education [4]. Children in India’s poorest households often live without adequate sanitation, [5] experience severe food insecurity and have poorer nutritional outcomes, [6–8] have access to lower quality health care facilities, [9] are less likely to receive care for severe diarrhea, [10] and are less likely to be fully immunized. [11–13] Maternal education is also associated with child health outcomes through a number of pathways. [14–16] The children of mothers who have no formal education are less likely to be vaccinated, [17] are more likely to experience growth faltering and malnutrition, [18, 19] are less likely to have been breastfed, [20] and are at a greater risk of mortality. [21]

Some previous studies have examined socioeconomic inequalities in child health throughout India. However, some of these studies are cross-sectional. For example, Porwal et al. use data from 2016 to 2018 to show persistent rich-poor gaps in child anthropometry throughout India. Other studies show socioeconomic trends over time, but only focus on a narrow set of child growth outcomes and do not use the most recent data. [22] Other studies show that disparities in child malnutrition either widened or stayed the same by household wealth and maternal education between 1993 and 2016. [22–27] Nguyen et al. showed that reductions in child anemia between 2006 and 2016 were largely driven by improvements in household wealth and maternal education. [28] In terms of child diarrhea and acute respiratory illness, much of the research examining trends over time consider these two outcomes as causes of death. [29–32] To our knowledge, there are no comprehensive studies that have examined the incidence or prevalence of these two outcomes over time by markers of SES in India.

Therefore, the purpose of this paper is to examine trends in under-five mortality, neonatal mortality, stunting, wasting, underweight, anemia, diarrhea, and acute respiratory illness between 1993 and 2021 by household wealth and maternal education. Additionally, unlike several previous studies, we examine the anemia and anthropometric failure outcomes based on severity. Examining the trends in all these outcomes over the past thirty-year period by household wealth and maternal education has not been done previously. Doing so is crucial given that India is currently not on track to meet many of the child health SDG targets by 2030. [1, 33, 34] Understanding which children, as a function of SES, are most vulnerable can help policy makers and program implementers better understand how to best target interventions for more equitable improvements in child health outcomes throughout India.

## Methods

### Data

We used data from five rounds of India’s National Family Health Survey (NFHS) to conduct this repeated cross-sectional analysis: NFHS-1, from 1992 to 1993, NFHS-2, from 1998 to 1999, NFHS-3, from 2005 to 2006, NFHS-4, from 2015 to 2016, and NFHS-5, from 2019 to 2021. Hereafter, we refer to the terminal year of the survey for simplicity. Each NFHS is designed to capture indicators of population health and nutrition, and each survey is representative at the household level. Households are selected via a two-stage sampling process. First, Primary Sampling Units (PSUs), which are villages in rural areas and wards in urban areas, were selected with probability proportional to size from districts within states. Households were then randomly selected from each PSU. This systematic approach for selecting households has been defined by the Demographic and Health Surveys Programme, and has been implemented in 90 different countries. [35]

### Study population

The NFHS survey contains data on key indicators of child health and wellbeing. However, the eligibility requirements have changed over time. Only NFHS-3, NFHS-4, and NFHS-5 asked the complete set of questions needed to diagnose ARI. Diarrhea data were collected for all living children born to a mother in the past four years in NFHS-1, any child born in the past three years in NFHS-2, and any child born in the past five years for NFHS-3, NFHS-4, and NFHS-5. Hemoglobin measurements were not taken in NFHS-1 but were taken for all living children between the ages of 6–35 months in NFHS-2, all living children under the age of five in NFHS-3 (except for the state of Nagaland), and all living children between the ages of 6–59 months in NFHS-4 and NFHS-5. Height and weight measurements were taken from all children under the age of four in NFHS-1 in every state except Tamil Nadu, West Bengal, Madhya Pradesh & Chhattisgarh, Andhra Pradesh & Telangana, and Himachal Pradesh. Anthropometric measurements were taken from all living children under the age of three in all states in NFHS-2, and for all children under the age of five in all states in NFHS-3, NFHS-4, and NFHS-5. Despite these varying age eligibility criteria, our analysis was conducted only on children between the ages of 0–36 months as this was the age range common to all five rounds of the survey. The final analytic sample, along with information on missingness, by outcome and survey round is presented in Table 1.

**Table 1:** Eligible, missing, and final analytic sample for each indicator of child health

**Survey**	Total kids under 36 months	Acute respiratory illness			Diarrhea			Anemia (mild/moderate/severe)			Stunting (moderate/severe)			Underweight (moderate/severe)			Wasting (moderate/severe)		
		Eligible	Missing/unknown	Final	Eligible	Missing/unknown	Final	Eligible	Missing/unknown/ implausible	Final	Eligible	Missing/unknown/ implausible	Final	Eligible	Missing/unknown/ implausible	Final	Eligible	Missing/unknown/ implausible	Final
NFHS 1	34,183	-	-	-	34,183	116	34,067	-	-	-	34,183	13,874	20,309	34,183	13,874	20,309	34,183	13,874	20,309
NFHS 2	28,662	-	-	-	28,662	80	28,582	23,583	3,053	20,530	28,662	3,841	24,821	28,662	3,841	24,821	28,662	3,841	24,821
NFHS 3	28,690	28,690	34	28,656	28,690	22	28,668	22,972	3,296	19,676	28,690	4,419	24,271	28,690	4,419	24,271	28,690	4,419	24,271
NFHS 4	145,299	145,299	81	145,218	145,299	143	145,156	122,649	7,553	115,096	145,299	13,025	132,274	145,299	13,025	132,274	145,299	13,025	132,274
NFHS 5	131,272	131,272	138	131,134	131,272	150	131,122	108,484	8,413	100,071	131,272	10,158	121,114	131,272	6,194	125,078	131,272	12,725	118,547

The following patterns of missingness were observed. Children in the lowest wealth quintile in NFHS-2 were slightly more likely to not have hemoglobin measurements than children in the highest wealth quintile. In NFHS-3, we found that children in the highest SES categories were less likely to not have hemoglobin measurements. This was true in NFHS-4 and NFHS-5 as well. For anthropometric outcomes, we found a small wealth difference between included and missing children. Those with missing data were more likely to be in a lower wealth quintile.

### Outcomes

We studied indicators of child morbidity and anthropometric failure. For indicators of morbidity, we examined the prevalence of child diarrhea (in the past two weeks), severe anemia (hemoglobin count less than 7.0 g/deciliter), moderate anemia (hemoglobin count between 7.0 and 9.9 g/deciliter), mild anemia (hemoglobin count between 10.0 and 10.9 g/deciliter), and acute respiratory illness. Hemoglobin values below 4 g/deciliter and above 18 g/deciliter were considered implausible, and those children were excluded from our analysis. Finally, we studied the prevalence of six forms of anthropometric failure. These were severe stunting (height-for-age Z score < -3 standard deviations of World Health Organization growth standards), moderate stunting (height-for-age Z score >= -3 and < -2 standard deviations of World Health Organization growth standards), severe underweight (weight-for-age Z score < -3 standard deviations of World Health Organization growth standards), moderate underweight (weight-for-age Z score >= -3 and < -2 standard deviations of World Health Organization growth standards), severe wasting (weight-for-height Z score < -3 standard deviations of World Health Organization growth standards), and moderate wasting (weight-for-height Z score >= -3 and < -2 standard deviations of World Health Organization growth standards). Each outcome in the three categories was dichotomized as yes/no for the purposes of this study. However, values above and below + 6 and − 6 standard deviations were considered implausible for height-for-age Z score. [36] Values above and below + 5 and − 5 standard deviations were considered implausible for weight-for-height Z score, and values above and below + 5 and − 6 standard deviations were considered implausible for weight-for-age Z score. [36] These children were excluded from our analysis.

### Statistical analysis

We estimated the weighted prevalence of each outcome in each survey round between 1993 and 2021, along with the standard errors, which is the square root of the variance divided by the sample size. These standard errors were then used to construct the 95% confidence intervals for each prevalence estimate. We estimated these values by household wealth quintile. In the NFHS, households are assigned a score, derived using a principal component analysis, based on consumer goods and assets they own (car, television, etc.), and their housing quality (toilet type, drinking water source, etc.). These scores are then assigned to each household member, who are then ranked nationally. These scores are then divided into five equal categories to create wealth quintiles. We also estimated the prevalence of each child health outcome by maternal education. The five categories of education we included were no schooling (zero years), 1st to 5th grade, 6th to 8th grade, 9th to 12th grade, and above 12th grade.

## Results

### Sample characteristics

Two-week prevalence of diarrhea was reported for 367,595 children across the five rounds. Hemoglobin data began being collected in 1999, and we used data from 255,373 children across four rounds of the NFHS to construct the anemia cutoffs. Data on ARI started being collected in 2006, and we used data from 305,008 children from three rounds of the NFHS. We estimated the prevalence of severe and moderate stunting by using height-for-age Z scores from 322,789 children across all five rounds. We estimated the prevalence of severe and moderate underweight by using weight-for-age Z scores from 326,753 children across all five rounds. Finally, we estimated the prevalence of severe and moderate wasting by using weight-for-height Z scores from 320,222 children across all five rounds. These results are presented in Table 1. Given that we used household wealth quintiles, approximately 20% of all children in each survey round were in each wealth quintile. In terms of mother’s education, the percent of mothers with no schooling decreased from 60% in 1993 to 22% in 2021. The percent of mothers with above 12th grade education increased from approximately 4% to nearly 14% over the same period.

### Changes in indicators of child health between 1993 and 2021

#### Indicators of morbidity

We found that the prevalence of ARI, diarrhea, moderate anemia, and severe anemia decreased among children in the lowest wealth quintile households and among children with mothers with no schooling between 1993 and 2021. Among children in the lowest wealth quintile households, we found that the prevalence of mild anemia increased from 23.6% (95% CI: 22.2–25.0) to 27.7% (95% CI: 27.1–28.2). Among children with mothers with no schooling, the prevalence of mild anemia increased from 22.3% (95% CI: 21.5–23.1) to 26.8% (95% CI: 26.2–27.4). Among children in the highest wealth quintile households, the prevalence of moderate anemia increased from 36.1% (95% CI: 34.7–37.6) to 39.3% (95% CI: 38.5–40.2), and the prevalence of mild anemia increased from 24.0% (95% CI: 22.7–25.4) to 28.4% (95% CI: 27.6–29.2). For children with mothers with above a 12th grade education, the prevalence of mild anemia increased from 22.0% (95% CI: 19.3–24.6) to 29.1% (95% CI: 28.3–29.8), and the prevalence of moderate anemia increased from 30.0% (95% CI: 27.1–32.9) to 37.2% (95% CI: 36.4–38.0). The prevalence of severe anemia increased from 1.6% (95% CI: 0.8–2.4) to 2.5% (95% CI: 2.2–2.8).

**Fig. 1:**
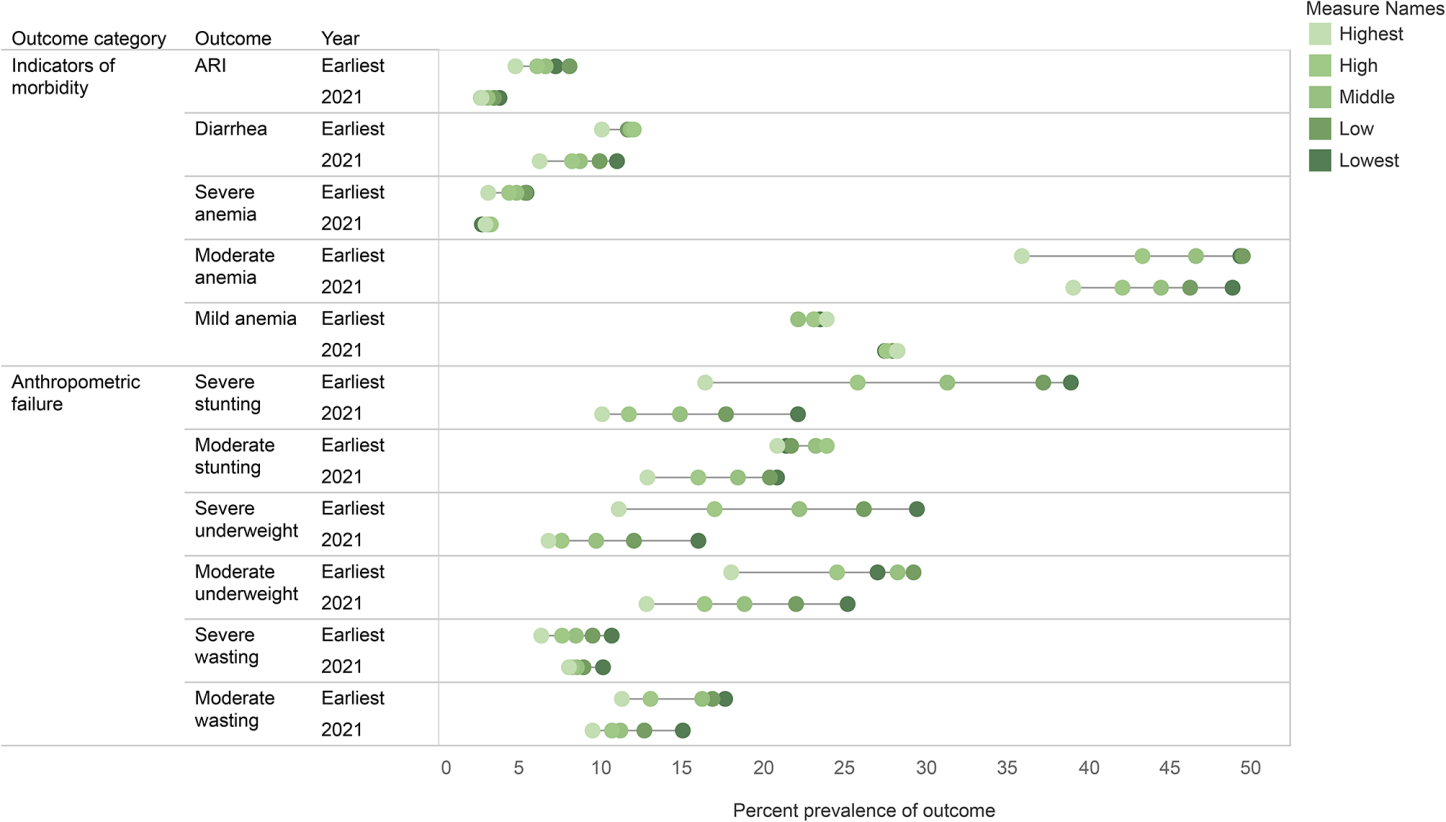
Prevalence of child health outcomes by household wealth, earliest year and 2021

**Fig. 2:**
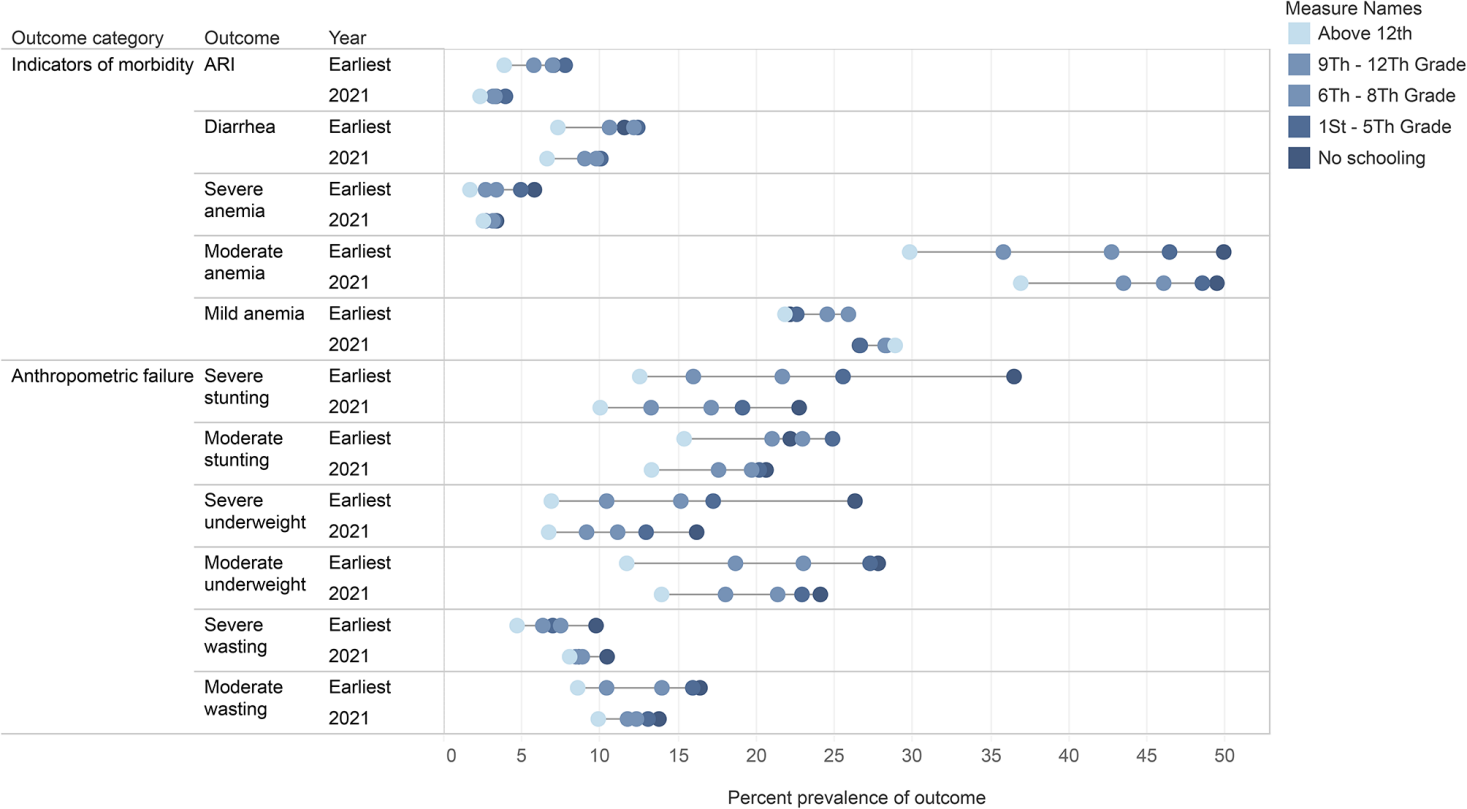
Prevalence of child health outcomes by maternal education, earliest year and 2021

Overall, the socioeconomic gradient for severe anemia and acute respiratory illness decreased between 1993 and 2021. This was true for household wealth and maternal education. However, the gap in the prevalence of diarrhea between children in the lowest and highest wealth quintiles increased between 1993 and 2021, while decreasing by mother’s education. The socioeconomic gradient for mild and moderate anemia increased between 1993 and 2021, and this was true for both household wealth and maternal education. These results are presented in Tables 2 and 3; Figs. 1 and 2, and supplementary Tables 1 and 2.

**Table 2:** Prevalence of child health outcomes (and confidence intervals) by year, lowest and highest wealth quintiles

	NFHS 1: 1992–1993		NFHS 2: 1998–1999		NFHS 3: 2005–2006		NFHS 4: 2015–2016		NFHS 5: 2019–2021	
	Lowest	Highest	Lowest	Highest	Lowest	Highest	Lowest	Highest	Lowest	Highest
ARI	-	-	-	-	7.2 [6.5–7.9]	4.7 [4.2–5.3]	3.6 [3.5–3.8]	2.3 [2.1–2.5]	3.7 [3.6–3.9]	2.6 [2.4–2.9]
Diarrhea	11.7 [10.9–12.5]	10.1 [9.4–10.8]	20.5 [19.4–21.6]	16.9 [15.9–17.9]	12.0 [11.1–12.9]	11.5 [10.7–12.3]	13.2 [12.9–13.6]	10.2 [9.8–10.7]	11.0 [10.7–11.4]	6.2 [5.9–6.6]
Severe anemia	-	-	5.4 [4.7–6.2]	3.0 [2.5–3.6]	3.6 [3-4.2]	2.8 [2.3–3.4]	2.1 [1.9–2.2]	2.0 [1.8–2.2]	2.7 [2.5–2.8]	2.9 [2.6–3.2]
Moderate anemia	-	-	49.7 [48.1–51.3]	36.1 [34.7–37.6]	55.8 [54.1–57.4]	38.1 [36.6–39.6]	41.0 [40.4–41.6]	30.7 [30.0-31.5]	49.2 [48.6–49.8]	39.3 [38.5–40.2]
Mild anemia	-	-	23.6 [22.2–25]	24.0 [22.7–25.4]	25.5 [24.1–27]	26.6 [25.2–27.9]	29.0 [28.5–29.6]	27.0 [26.3–27.7]	27.7 [27.1–28.2]	28.4 [27.6–29.2]
Severe stunting	39.2 [37.5–40.8]	16.5 [15.4–17.6]	38.8 [37.3–40.2]	12.1 [11.2–13]	32.2 [30.8–33.7]	7.5 [6.7–8.2]	24.6 [24.1–25]	8.6 [8.2-9.0]	22.3 [21.8–22.7]	10.1 [9.6–10.6]
Moderate stunting	21.5 [20.1–22.9]	21.0 [19.8–22.2]	23.6 [22.3–24.9]	19.8 [18.7–20.9]	24.0 [22.7–25.3]	17.1 [16.1–18.1]	23.0 [22.5–23.4]	13.4 [12.9–13.9]	21.0 [20.5–21.4]	12.9 [12.4–13.4]
Severe underweight	29.6 [28.1–31.2]	11.1 [10.2–12.0]	28.1 [26.7–29.4]	5.7 [5.1–6.4]	25.6 [24.2–26.9]	4.2 [3.6–4.7]	17.7 [17.3–18.1]	5.1 [4.8–5.4]	16.1 [15.7–16.5]	6.8 [6.4–7.2]
Moderate underweight	27.2 [25.7–28.7]	18.1 [17.0-19.2]	29.3 [27.9–30.7]	16.4 [15.3–17.4]	29.8 [28.4–31.2]	13.4 [12.5–14.4]	29.4 [28.9–29.9]	14.1 [13.6–14.6]	25.3 [24.9–25.8]	12.9 [12.4–13.4]
Severe wasting	10.7 [9.7–11.8]	6.3 [5.6-7.0]	9.8 [8.9–10.7]	4.3 [3.7–4.8]	10.7 [9.8–11.7]	4.6 [4.0-5.1]	10.6 [10.2–10.9]	7.5 [7.1–7.9]	10.2 [9.8–10.5]	8.1 [7.6–8.5]
Moderate wasting	17.7 [16.4–19.0]	11.3 [10.4–12.3]	17.1 [16.0-18.3]	8.0 [7.2–8.7]	18.9 [17.7–20.1]	9.2 [8.4–10.0]	17.5 [17.1–17.9]	11.9 [11.4–12.3]	15.1 [14.7–15.5]	9.5 [9.1–10.0]

**Fig. 3:**
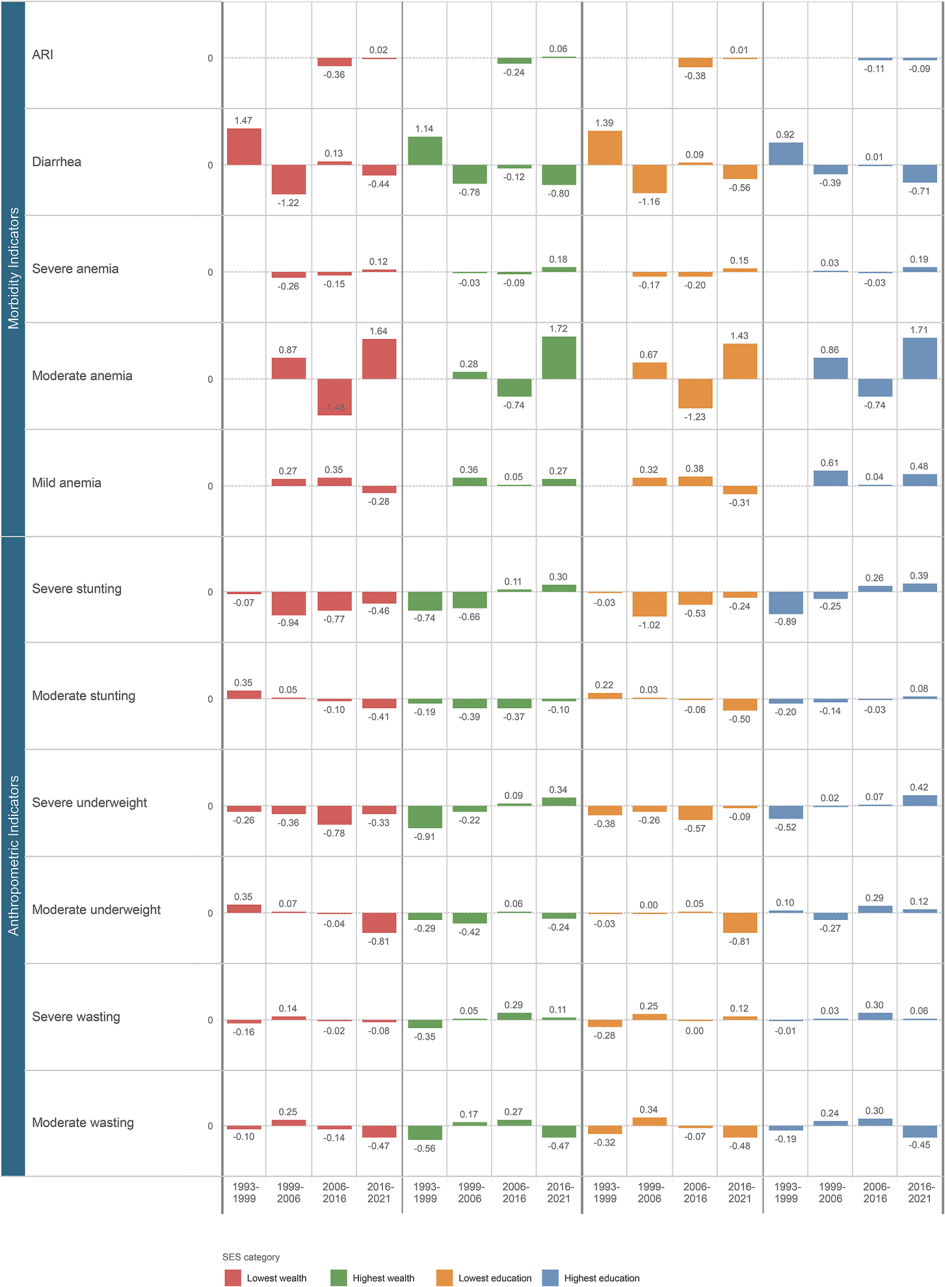
Standardized annual change (SAC) in percentage points by outcome for each time period. Values were derived by subtracting the prevalence in year 1 from the prevalence in year 2 and then dividing by the number of intervening years. Red denotes SAC for lowest wealth quintile, green for highest wealth quintile, orange for no schooling, and dark blue for above 12th grade education

We found that the largest declines in prevalence for diarrhea, severe anemia, and ARI occurred prior to 2016. However, the prevalence of severe anemia increased between 2016 and 2021 for all children, regardless of household wealth or maternal education. The overall prevalence of moderate anemia increased between 1993 and 2021, with the largest increases occurring between 2016 and 2021 regardless of household wealth or maternal education. Similarly, the overall prevalence of mild anemia also increased between 1993 and 2021. However, the largest increases occurred prior to 2016. These results are presented in Fig. 3.

As of 2021, we found that the absolute inequality in the prevalence between the lowest and highest wealth quintile children for ARI, diarrhea, severe anemia, moderate anemia, and mild anemia was greater in urban communities than rural communities. Except for ARI, the same was true when comparing children whose mothers had no schooling versus those whose mothers had above a 12th grade education. These results are presented in supplementary Tables 3–6.

#### Anthropometric failure

We found that the prevalence of severe stunting, moderate stunting, severe underweight, moderate underweight, severe wasting, and moderate wasting all decreased among children in the lowest wealth quintiles households between 1993 and 2021. We found a similar pattern when examining children with mothers with no schooling. The only exception to this was that the prevalence of severe wasting increased from 9.8% (95% CI: 9.2–10.3) to 10.5% (95% CI 10.1–10.9). Among children in the highest wealth quintile households, the prevalence of severe wasting increased from 6.3% (95% CI: 5.6-7.0) to 8.1 (95% CI: 7.6–8.5). Among children with mothers with above a 12th grade education, the prevalence of moderate underweight increased from 11.8% (95% CI: 9.6–13.9) to 14.0% (95% CI:13.5–14.5) The prevalence of severe wasting increased from 4.7% (95% CI: 3.2–6.1) to 8.1% (95% CI: 7.7–8.5), and the prevalence of moderate wasting increased from 8.6% (95% CI: 6.7–10.5) to 9.9% (95% CI: 9.5–10.4).

Overall, the household wealth gradient decreased for severe stunting, severe underweight, severe wasting, and moderate wasting. The household wealth gradient increased for moderate stunting and moderate underweight. The maternal education gradient decreased for severe stunting, severe underweight, moderate underweight, severe wasting, and moderate wasting. The maternal education gradient increased for moderate stunting. These results are presented in Tables 2 and 3; Figs. 1 and 2, and supplementary Tables 1 and 2.

**Table 3:** Prevalence of child health outcomes (and confidence intervals) by year, no schooling and above 12th grade education for mothers

	NFHS 1: 1992–1993		NFHS 2: 1998–1999		NFHS 3: 2005–2006		NFHS 4: 2015–2016		NFHS 5: 2019–2021	
	No schooling	Above 12th grade	No schooling	Above 12th grade	No schooling	Above 12th grade	No schooling	Above 12th grade	No schooling	Above 12th grade
ARI	-	-	-	-	7.0 [6.6–7.5]	3.8 [3.1–4.6]	3.3 [3.1–3.4]	2.7 [2.5-3]	3.3 [3.1–3.5]	2.3 [2.1–2.5]
Diarrhea	11.6 [11.2–12.1]	7.3 [5.9–8.7]	20.0 [19.3–20.6]	12.8 [11-14.6]	11.8 [11.2–12.4]	10.1 [8.9–11.3]	12.8 [12.4–13.1]	10.2 [9.7–10.6]	10.0 [9.6–10.3]	6.6 [6.3-7]
Severe anemia	-	-	5.8 [5.3–6.3]	1.6 [0.8–2.4]	4.6 [4.1-5]	1.9 [1.2–2.6]	2.6 [2.4–2.8]	1.5 [1.3–1.8]	3.4 [3.1–3.6]	2.5 [2.2–2.8]
Moderate anemia	-	-	50.3 [49.3–51.3]	30.0 [27.1–32.9]	54.9 [53.8–56.1]	36.0 [33.6–38.4]	42.7 [42.2–43.2]	28.6 [27.8–29.5]	49.8 [49.2–50.5]	37.2 [36.4–38.0]
Mild anemia	-	-	22.3 [21.5–23.1]	22.0 [19.3–24.6]	24.5 [23.6–25.5]	26.3 [24.1–28.5]	28.3 [27.9–28.8]	26.7 [25.9–27.5]	26.8 [26.2–27.4]	29.1 [28.3–29.8]
Severe stunting	36.7 [35.9–37.6]	12.6 [10.3–14.9]	36.6 [35.7–37.4]	7.3 [5.8–8.7]	29.4 [28.5–30.3]	5.5 [4.5–6.5]	24.1 [23.7–24.5]	8.1 [7.6–8.6]	22.9 [22.4–23.4]	10.0 [9.6–10.5]
Moderate stunting	22.3 [21.5–23.1]	15.5 [13-17.9]	23.7 [22.9–24.4]	14.3 [12.3–16.3]	23.8 [23-24.7]	13.3 [11.8–14.8]	23.3 [22.8–23.7]	13.0 [12.4–13.5]	20.7 [20.2–21.3]	13.4 [12.9–13.9]
Severe underweight	26.5 [25.7–27.3]	6.9 [5.2–8.6]	24.2 [23.4–25.0]	3.8 [2.7–4.9]	22.4 [21.6–23.3]	3.9 [3.1–4.8]	16.7 [16.3–17.1]	4.6 [4.3-5.0]	16.3 [15.8–16.7]	6.7 [6.4–7.1]
Moderate underweight	28 [27.2–28.8]	11.8 [9.6–13.9]	27.8 [27.0-28.6]	12.4 [10.5–14.2]	27.8 [26.9–28.7]	10.5 [9.1–11.8]	28.3 [27.9–28.8]	13.4 [12.8–14]	24.3 [23.7–24.8]	14.0 [13.5–14.5]
Severe wasting	9.8 [9.2–10.3]	4.7 [3.2–6.1]	8.1 [7.6–8.6]	4.6 [3.4–5.8]	9.9 [9.3–10.5]	4.8 [3.9–5.8]	9.9 [9.6–10.2]	7.8 [7.3–8.2]	10.5 [10.1–10.9]	8.1 [7.7–8.5]
Moderate wasting	16.5 [15.8–17.2]	8.6 [6.7–10.5]	14.6 [13.9–15.2]	7.4 [5.9–8.9]	16.9 [16.2–17.7]	9.1 [7.9–10.4]	16.2 [15.9–16.6]	12.2 [11.6–12.7]	13.8 [13.4–14.3]	9.9 [9.5–10.4]

For children in the lowest SES groups, we found that the largest declines in severe stunting, severe underweight, and severe wasting occurred prior to 2016, while the largest declines in moderate stunting, moderate underweight, and moderate wasting occurred between 2016 and 2021. For children in the highest SES categories, the largest declines in prevalence of all forms of anthropometric failure occurred before 2016. There were two exceptions to this, both for children with mothers with no schooling. First, the prevalence of severe wasting has only increased, with the largest increase occurring between 2006 and 2016. For moderate wasting, the largest decline came between 2016 and 2021. These results are presented in Fig. 3.

As of 2021, we found that the absolute inequality between children in the lowest and highest wealth quintiles was higher in rural communities than urban communities for severe stunting, severe underweight, severe wasting, and moderate wasting. The absolute inequality between children whose mothers had no schooling and those whose mothers had above a 12th grade education was higher in rural communities than urban communities for all the anthropometric outcomes except for moderate underweight. These results are presented in supplementary Tables 3–6.

## Discussion

Our study had several salient findings. First, when looking at household wealth, the largest reductions in outcome prevalence were among children in the poorest households. The exceptions to this were for diarrhea, moderate stunting, and moderate underweight. Similarly, the largest reductions in outcome prevalence were among children with mothers with no education except for moderate stunting. Second, the wealth gradient decreased for ARI, severe anemia, severe stunting, severe underweight, severe wasting, and moderate wasting. The maternal education gradient decreased for ARI, diarrhea, severe anemia, severe stunting, severe underweight, moderate underweight, severe wasting, and moderate wasting. Third, we found that most of the improvements in indicators of mortality, morbidity, and anthropometric failure occurred before 2016.

There are several data limitations to this study. First, diarrhea and ARI are reported by the mothers, and thus subject to recall bias. Nevertheless, these outcomes have been used in prior publications given the overall high-quality of the data. Second, child anthropometry was not measured in all states during NFHS-1 due to a lack of measurement equipment. Therefore, the results for these outcomes from NFHS-1 is not representative of all children. Third, data collection for NFHS-5 began in 2019, but was disrupted by COVID-19, before being completed in 2021. The extent to which this disruption is associated with any bias in responses is unknown, as is the full effect of the pandemic on the outcomes included in this study.

Over the last few decades, India’s government has launched several programs aimed at improving child health. Many of these programs have had a pro-poor focus. For example, various initiatives under India’s Integrated Child Development Services program and the National Health Mission were designed to improve child morbidity and nutrition outcomes. Our results show that the largest improvements in child health outcomes between 1993 and 2021 were largely concentrated among children in the lowest SES categories. While our study does not present a causal analysis, it is possible that these improvements are in part due to various pro-poor programs aimed at improving maternal and child health. This would be consistent with prior studies which show how many of these programs have led to significant improvements in child health. [37] However, our results show that for outcomes such as moderate stunting, children in the highest SES groups experienced the greatest reductions in prevalence between 1993 and 2021. This could be due to myriad factors including a complex set of social and economic barriers to healthcare access, [38] or because programs aimed at improving child health often do not reach the children who need them the most. [39] Our results once again emphasize the urgent need for programs that target and reach India’s most vulnerable children in order to prevent adverse outcomes such as diarrhea and stunting. In addition to prioritizing socioeconomically vulnerable children, programs should be geographically targeted. Doing so is critical given that there is considerable geographic variation in the prevalence of diarrhea, [40] ARI, [41] anemia, [42] and anthropometric failure across India. [43]

Our results also highlight how some inequalities have worsened over time. For instance, the gap in diarrhea prevalence between children in the lowest and highest wealth quintiles widened between 1993 and 2021. This widening is attributable to the fact that the absolute decrease in the prevalence of diarrhea was greater among children in the highest wealth quintile than children in the lowest wealth quintile. Microbial contamination and diarrhea risk fluctuate by season, [44] something our analysis could not account for. Nevertheless, the pace of reduction was faster among children in the highest SES groups than those in the lowest between 2016 and 2021. This worsening inequality comes despite significant improvements in water and sanitation coverage throughout India over the past few decades. [5, 45] However, progress towards improving water and sanitation coverage has been slowest among the most socioeconomically marginalized households, especially those in rural areas. [5, 45, 46] Models from India show that the benefits of improved access to water and sanitation on diarrhea are highly progressive in that their impact on poor households is greater. [47] Thus, alleviating the socioeconomic gradient in diarrhea prevalence will require pro-poor policies for improving access to water and sanitation.

Finally, our results underscore the importance of stratifying child health outcomes by severity and SES categories. A prior study shows that the prevalence of any stunting has increased among children in the two highest wealth quintiles between 2006 and 2021. [48] Our results, however, show that this increase over the past 15 years is being driven by increases in severe stunting among children in the two highest wealth quintiles. This is also true for severe wasting. And for children who are moderately underweight, we show that the increases are among children with mothers with higher education. Singh et al. also highlight that while the prevalence of mild, moderate and severe anemia steadily decreased among children between 1999 and 2016, it actually increased between 2016 and 2021. [49] Our results confirm these findings. However, we show that the wealth gradient for severe anemia is extremely low now with a very small difference between children in the lowest and highest wealth quintiles. This gradient is larger when examining the outcome by maternal education. We also show that the prevalence of moderate anemia has increased regardless of wealth or maternal education. This could be due to a mother’s anemia status, various socioeconomic factors, the number of children born to a mother, and persistent micronutrient deficiencies. [49–51] On the other hand, the increases in mild anemia have largely been among children in the highest wealth quintile homes and among children with mothers with higher education. This could be due to factors such as when the survey was conducted given seasonal variations in anemia. [49] Thus, our results further highlight the importance of examining child health outcomes by socioeconomic status.

In conclusion, our paper comprehensively examined the prevalence trends of 11 different child health outcomes by household wealth and maternal education, two markers of SES that are associated with child health between 1993 and 2021. We show that the health of India’s poorest children has improved over this time, and that health disparities between the lowest and highest SES groups have narrowed for many outcomes. Yet in some cases, this reduction in disparity was because the prevalence had increased among children in the highest SES groups. Pro-poor policies are vital given that India’s most marginalized children still have the highest prevalence of 11 different outcomes. But policy makers must mitigate any further declines in health among India’s better-off children, too.

### Electronic supplementary material

Below is the link to the electronic supplementary material.

Supplementary Material 1

## Data Availability

The data that support the findings of this study are openly available through the Demographic and Health Surveys program website: https://dhsprogram.com/Countries/Country-Main.cfm?ctry_id=57&c=India.
