# Supplementary material for: Socioeconomic inequality in child health outcomes in India: analyzing trends between 1993 and 2021
Source: Int J Equity Health. 2024 Jul 31;23:149. doi: 10.1186/s12939-024-02218-z (PMC11290299; doi:10.1186/s12939-024-02218-z)
Supplement: Supplementary file 1 — Supplementary Material 1 [file 12939_2024_2218_MOESM1_ESM.docx]

**Supplementary Online Content**

Jain A, Kim R, Swaminathan S, Subramanian SV. Socioeconomic inequality in child health outcomes in India: analyzing trends between 1993 and 2021. *International Journal for Equity in Health.* 2024.

**Supplemental table 1:** Prevalence (standard error) for each outcome by each wealth quintile, 1993, 1999, 2006, 2016, and 2021

**Supplemental table 2:** Prevalence (standard error) for each outcome by each category of maternal education, 1993, 1999, 2006, 2016, and 2021

**Supplemental table 3:** Prevalence (95%) for each outcome by lowest and highest wealth quintile, 1993, 1999, 2006, 2016, and 2021 in urban communities

**Supplemental table 4:** Prevalence (95%) for each outcome by lowest and highest wealth quintile, 1993, 1999, 2006, 2016, and 2021 in rural communities

**Supplemental table 5:** Prevalence (95%) for each outcome by lowest and highest category of maternal education, 1993, 1999, 2006, 2016, and 2021 in urban communities

**Supplemental table 6:** Prevalence (95%) for each outcome by lowest and highest category of maternal education, 1993, 1999, 2006, 2016, and 2021 in rural communities

Supplemental table 1: Prevalence (standard error) for each outcome by each wealth quintile, 1993, 1999, 2006, 2016, and 2021

|  | NFHS 1: 1992–1993 | | | | | NFHS 2: 1998–1999 | | | | | | NFHS 3: 2005–2006 | | | | | | NFHS 4: 2015–2016 | | | | | | NFHS 5: 2019–2021 | | | | | |
| --- | --- | --- | --- | --- | --- | --- | --- | --- | --- | --- | --- | --- | --- | --- | --- | --- | --- | --- | --- | --- | --- | --- | --- | --- | --- | --- | --- | --- | --- |
|  | Lowest | Low | Middle | High | Highest | Lowest | Low | Middle | High | Highest | Lowest | | Low | Middle | High | Highest | Lowest | | Low | Middle | High | Highest | Lowest | | Low | Middle | High | Highest |  |
| ARI | - | - | - | - | - | - | - | - | - | - | 7.2 (0.4) | | 8.1 (0.4) | 6.6 (0.3) | 6.1 (0.3) | 4.7 (0.3) | 3.6 (0.1) | | 3.3 (0.1) | 3 (0.1) | 2.8 (0.1) | 2.3 (0.1) | 3.7 (0.1) | | 3.4 (0.1) | 3 (0.1) | 2.6 (0.1) | 2.6 (0.1) |  |
| Diarrhea | 11.7 (0.4) | 11.7 (0.4) | 11.9 (0.4) | 12.1 (0.4) | 10.1 (0.4) | 20.5 (0.6) | 18.7 (0.5) | 20.2 (0.5) | 18.1 (0.5) | 16.9 (0.5) | 12 (0.5) | | 12.3 (0.5) | 12.3 (0.4) | 12.9 (0.4) | 11.5 (0.4) | 13.2 (0.2) | | 12.4 (0.2) | 12.1 (0.2) | 11 (0.2) | 10.2 (0.2) | 11 (0.2) | | 10 (0.2) | 8.7 (0.2) | 8.2 (0.2) | 6.2 (0.2) |  |
| Severe anemia | - | - | - | - | - | 5.4 (0.4) | 5.4 (0.4) | 4.8 (0.3) | 4.3 (0.3) | 3 (0.3) | 3.6 (0.3) | | 4.4 (0.3) | 4.5 (0.3) | 3.2 (0.3) | 2.8 (0.3) | 2.1 (0.1) | | 2.1 (0.1) | 2.3 (0.1) | 2.1 (0.1) | 2 (0.1) | 2.7 (0.1) | | 2.9 (0.1) | 3 (0.1) | 3.2 (0.1) | 2.9 (0.1) |  |
| Moderate anemia | - | - | - | - | - | 49.7 (0.8) | 49.8 (0.8) | 46.9 (0.8) | 43.6 (0.7) | 36.1 (0.8) | 55.8 (0.8) | | 52.9 (0.8) | 49.4 (0.8) | 47 (0.8) | 38.1 (0.8) | 41 (0.3) | | 37.1 (0.3) | 36.6 (0.3) | 33.9 (0.3) | 30.7 (0.4) | 49.2 (0.3) | | 46.6 (0.3) | 44.8 (0.4) | 42.4 (0.4) | 39.3 (0.4) |  |
| Mild anemia | - | - | - | - | - | 23.6 (0.7) | 23.3 (0.7) | 22.3 (0.6) | 23.2 (0.6) | 24 (0.7) | 25.5 (0.7) | | 25.8 (0.7) | 25.2 (0.7) | 25.5 (0.7) | 26.6 (0.7) | 29 (0.3) | | 29.3 (0.3) | 28.2 (0.3) | 27.3 (0.3) | 27 (0.4) | 27.7 (0.3) | | 28.1 (0.3) | 28.4 (0.3) | 27.7 (0.3) | 28.4 (0.4) |  |
| Severe stunting | 39.2 (0.8) | 37.5 (0.8) | 31.5 (0.8) | 26 (0.6) | 16.5 (0.5) | 38.8 (0.7) | 34.2 (0.7) | 28.7 (0.6) | 21 (0.5) | 12.1 (0.5) | 32.2 (0.7) | | 26 (0.7) | 21.3 (0.6) | 15.9 (0.5) | 7.5 (0.4) | 24.6 (0.2) | | 18.1 (0.2) | 13.6 (0.2) | 11.4 (0.2) | 8.6 (0.2) | 22.3 (0.2) | | 17.8 (0.2) | 14.9 (0.2) | 11.8 (0.2) | 10.1 (0.2) |  |
| Moderate stunting | 21.5 (0.7) | 21.8 (0.7) | 23.4 (0.7) | 24 (0.6) | 21 (0.6) | 23.6 (0.6) | 24.8 (0.6) | 23.9 (0.6) | 23.4 (0.6) | 19.8 (0.6) | 24 (0.7) | | 25 (0.7) | 23.7 (0.6) | 22.2 (0.6) | 17.1 (0.5) | 23 (0.2) | | 22.4 (0.2) | 20.9 (0.2) | 17 (0.3) | 13.4 (0.3) | 21 (0.2) | | 20.5 (0.2) | 18.5 (0.3) | 16.1 (0.3) | 12.9 (0.3) |  |
| Severe underweight | 29.6 (0.8) | 26.3 (0.8) | 22.3 (0.7) | 17.1 (0.5) | 11.1 (0.5) | 28.1 (0.7) | 22.4 (0.6) | 17.6 (0.5) | 11.4 (0.4) | 5.7 (0.3) | 25.6 (0.7) | | 20.3 (0.6) | 13.1 (0.5) | 9.2 (0.4) | 4.2 (0.3) | 17.7 (0.2) | | 12.3 (0.2) | 8.5 (0.2) | 6.6 (0.2) | 5.1 (0.2) | 16.1 (0.2) | | 12.1 (0.2) | 9.7 (0.2) | 7.6 (0.2) | 6.8 (0.2) |  |
| Moderate underweight | 27.2 (0.8) | 29.4 (0.8) | 28.4 (0.7) | 24.7 (0.6) | 18.1 (0.6) | 29.3 (0.7) | 27.7 (0.7) | 26.8 (0.6) | 23.2 (0.6) | 16.4 (0.5) | 29.8 (0.7) | | 28 (0.7) | 25.6 (0.6) | 21.5 (0.6) | 13.4 (0.5) | 29.4 (0.2) | | 25.4 (0.2) | 22.3 (0.3) | 18.9 (0.3) | 14.1 (0.3) | 25.3 (0.2) | | 22.1 (0.2) | 18.9 (0.3) | 16.5 (0.3) | 12.9 (0.3) |  |
| Severe wasting | 10.7 (0.5) | 9.5 (0.5) | 8.5 (0.5) | 7.6 (0.4) | 6.3 (0.4) | 9.8 (0.5) | 7.6 (0.4) | 6.5 (0.3) | 4.6 (0.3) | 4.3 (0.3) | 10.7 (0.5) | | 9 (0.4) | 7.4 (0.4) | 6.1 (0.3) | 4.6 (0.3) | 10.6 (0.2) | | 9.2 (0.2) | 8.2 (0.2) | 7.7 (0.2) | 7.5 (0.2) | 10.2 (0.2) | | 9 (0.2) | 8.5 (0.2) | 8.2 (0.2) | 8.1 (0.2) |  |
| Moderate wasting | 17.7 (0.7) | 17 (0.6) | 16.3 (0.6) | 13.1 (0.5) | 11.3 (0.5) | 17.1 (0.6) | 14.3 (0.5) | 13.2 (0.5) | 11.1 (0.4) | 8 (0.4) | 18.9 (0.6) | | 17 (0.6) | 14.2 (0.5) | 12.4 (0.4) | 9.2 (0.4) | 17.5 (0.2) | | 15.1 (0.2) | 13.8 (0.2) | 12.9 (0.2) | 11.9 (0.2) | 15.1 (0.2) | | 12.7 (0.2) | 11.2 (0.2) | 10.7 (0.2) | 9.5 (0.2) |  |

Supplemental table 2: Prevalence (standard error) for each outcome by each category of maternal education, 1993, 1999, 2006, 2016, and 2021

|  | NFHS 1: 1992–1993 | | | | | NFHS 2: 1998–1999 | | | | | NFHS 3: 2005–2006 | | | | | NFHS 4: 2015–2016 | | | | | NFHS 5: 2019–2021 | | | | |
| --- | --- | --- | --- | --- | --- | --- | --- | --- | --- | --- | --- | --- | --- | --- | --- | --- | --- | --- | --- | --- | --- | --- | --- | --- | --- |
|  | None | 1st-5th | 6th-8th | 9th-12th | 12th + | None | 1st-5th | 6th-8th | 9th-12th | 12th + | None | 1st-5th | 6th-8th | 9th-12th | 12th + | None | 1st-5th | 6th-8th | 9th-12th | 12th + | None | 1st-5th | 6th-8th | 9th-12th | 12th + |
| ARI | - | - | - | - | - | - | - | - | - | - | 7 (0.2) | 7.8 (0.4) | 7 (0.4) | 5.8 (0.3) | 3.8 (0.4) | 3.3 (0.1) | 3.6 (0.1) | 3.4 (0.1) | 2.6 (0.1) | 2.7 (0.1) | 3.3 (0.1) | 3.9 (0.2) | 3.3 (0.1) | 3.1 (0.1) | 2.3 (0.1) |
| Diarrhea | 11.6 (0.2) | 12.5 (0.5) | 12.2 (0.5) | 10.6 (0.5) | 7.3 (0.7) | 20 (0.3) | 19.9 (0.6) | 18.6 (0.6) | 16.7 (0.5) | 12.8 (0.9) | 11.8 (0.3) | 12.6 (0.5) | 13.1 (0.5) | 12.9 (0.4) | 10.1 (0.6) | 12.8 (0.2) | 12.9 (0.2) | 12.3 (0.2) | 11.3 (0.2) | 10.2 (0.2) | 10 (0.2) | 10.1 (0.2) | 9.8 (0.2) | 9 (0.1) | 6.6 (0.2) |
| Severe anemia | - | - | - | - | - | 5.8 (0.2) | 4.9 (0.4) | 3.3 (0.3) | 2.6 (0.3) | 1.6 (0.4) | 4.6 (0.2) | 4 (0.4) | 3.2 (0.3) | 2.5 (0.2) | 1.9 (0.3) | 2.6 (0.1) | 2.3 (0.1) | 2.1 (0.1) | 1.8 (0.1) | 1.5 (0.1) | 3.4 (0.1) | 3.2 (0.2) | 3.1 (0.1) | 2.7 (0.1) | 2.5 (0.1) |
| Moderate anemia | - | - | - | - | - | 50.3 (0.5) | 46.8 (0.9) | 43 (0.9) | 36.1 (0.8) | 30 (1.5) | 54.9 (0.6) | 49.8 (1) | 46.2 (0.9) | 42.7 (0.7) | 36 (1.2) | 42.7 (0.3) | 38.8 (0.4) | 35.8 (0.3) | 32.7 (0.3) | 28.6 (0.4) | 49.8 (0.3) | 48.9 (0.5) | 46.4 (0.4) | 43.8 (0.3) | 37.2 (0.4) |
| Mild anemia | - | - | - | - | - | 22.3 (0.4) | 22.7 (0.7) | 24.7 (0.8) | 26.1 (0.7) | 22 (1.3) | 24.5 (0.5) | 25.6 (0.8) | 27.2 (0.8) | 27.2 (0.7) | 26.3 (1.1) | 28.3 (0.2) | 28.9 (0.4) | 28.6 (0.3) | 28.5 (0.2) | 26.7 (0.4) | 26.8 (0.3) | 26.8 (0.4) | 28.5 (0.3) | 28.4 (0.2) | 29.1 (0.4) |
| Severe stunting | 36.7 (0.5) | 25.7 (0.8) | 21.8 (0.8) | 16.1 (0.7) | 12.6 (1.2) | 36.6 (0.4) | 25.1 (0.7) | 19.5 (0.7) | 13.8 (0.5) | 7.3 (0.8) | 29.4 (0.5) | 22.3 (0.7) | 18.3 (0.6) | 10.5 (0.4) | 5.5 (0.5) | 24.1 (0.2) | 18.1 (0.3) | 15.3 (0.2) | 11.4 (0.2) | 8.1 (0.2) | 22.9 (0.3) | 19.2 (0.3) | 17.2 (0.3) | 13.3 (0.2) | 10 (0.2) |
| Moderate stunting | 22.3 (0.4) | 25 (0.8) | 23.1 (0.8) | 21.1 (0.7) | 15.5 (1.3) | 23.7 (0.4) | 25.3 (0.7) | 24.2 (0.7) | 21.4 (0.6) | 14.3 (1) | 23.8 (0.4) | 23.9 (0.7) | 24.2 (0.7) | 21 (0.5) | 13.3 (0.8) | 23.3 (0.2) | 22.8 (0.3) | 20.9 (0.3) | 17.6 (0.2) | 13 (0.3) | 20.7 (0.3) | 20.3 (0.3) | 19.8 (0.3) | 17.7 (0.2) | 13.4 (0.3) |
| Severe underweight | 26.5 (0.4) | 17.3 (0.7) | 15.2 (0.7) | 10.5 (0.6) | 6.9 (0.9) | 24.2 (0.4) | 14.8 (0.6) | 11.4 (0.5) | 7.8 (0.4) | 3.8 (0.6) | 22.4 (0.4) | 14.9 (0.6) | 10.7 (0.5) | 6.5 (0.3) | 3.9 (0.4) | 16.7 (0.2) | 11.9 (0.2) | 10.3 (0.2) | 7.3 (0.1) | 4.6 (0.2) | 16.3 (0.2) | 13 (0.3) | 11.2 (0.2) | 9.2 (0.1) | 6.7 (0.2) |
| Moderate underweight | 28 (0.4) | 27.5 (0.9) | 23.2 (0.8) | 18.8 (0.7) | 11.8 (1.1) | 27.8 (0.4) | 28.1 (0.7) | 22.8 (0.7) | 18.4 (0.6) | 12.4 (1) | 27.8 (0.5) | 25.8 (0.7) | 25.3 (0.7) | 19.2 (0.5) | 10.5 (0.7) | 28.3 (0.2) | 25.8 (0.3) | 23.8 (0.3) | 19.6 (0.2) | 13.4 (0.3) | 24.3 (0.3) | 23.1 (0.3) | 21.5 (0.3) | 18.1 (0.2) | 14 (0.3) |
| Severe wasting | 9.8 (0.3) | 7 (0.5) | 7.5 (0.5) | 6.3 (0.4) | 4.7 (0.7) | 8.1 (0.2) | 6 (0.4) | 5.2 (0.4) | 4.4 (0.3) | 4.6 (0.6) | 9.9 (0.3) | 7.4 (0.4) | 6.8 (0.4) | 5 (0.3) | 4.8 (0.5) | 9.9 (0.2) | 8.9 (0.2) | 9.1 (0.2) | 8 (0.1) | 7.8 (0.2) | 10.5 (0.2) | 8.7 (0.2) | 8.9 (0.2) | 8.5 (0.1) | 8.1 (0.2) |
| Moderate wasting | 16.5 (0.3) | 16 (0.7) | 14 (0.7) | 10.5 (0.6) | 8.6 (1) | 14.6 (0.3) | 13 (0.5) | 12.1 (0.6) | 10 (0.5) | 7.4 (0.8) | 16.9 (0.4) | 15.6 (0.6) | 13.9 (0.6) | 11.5 (0.4) | 9.1 (0.6) | 16.2 (0.2) | 15 (0.3) | 14.7 (0.2) | 13.5 (0.2) | 12.2 (0.3) | 13.8 (0.2) | 13.1 (0.3) | 12.4 (0.2) | 11.8 (0.2) | 9.9 (0.2) |

Supplemental table 3: Prevalence (95%) for each outcome by lowest and highest wealth quintile, 1993, 1999, 2006, 2016, and 2021 in urban communities

|  | NFHS 1: 1992–1993 | | NFHS 2: 1998–1999 | | NFHS 3: 2005–2006 | | NFHS 4: 2015–2016 | | NFHS 5: 2019–2021 | |
| --- | --- | --- | --- | --- | --- | --- | --- | --- | --- | --- |
|  | Lowest | Highest | Lowest | Highest | Lowest | Highest | Lowest | Highest | Lowest | Highest |
| ARI | - | - | - | - | 7.4 [4.7–10.1] | 4.8 [4.2–5.4] | 3.1 [2.3-4] | 2.1 [1.8–2.3] | 3.2 [2.2–4.2] | 2.3 [2-2.6] |
| Diarrhea | 6.2 [2.5–10] | 9.6 [8.8–10.4] | 24.1 [18.1–30.1] | 17 [15.8–18.3] | 12.8 [9.3–16.3] | 11.4 [10.5–12.3] | 11.6 [10.1–13.1] | 9.9 [9.4–10.4] | 13.1 [11.2–15.1] | 6 [5.5–6.4] |
| Severe anemia | - | - | 4.1 [0.9–7.2] | 2.8 [2.2–3.5] | 7.9 [4.4–11.4] | 2.9 [2.3–3.5] | 3 [2-3.9] | 2 [1.7–2.2] | 3.3 [2.1–4.4] | 2.9 [2.5–3.3] |
| Moderate anemia | - | - | 57.5 [49.7–65.3] | 35.7 [33.9–37.5] | 53.1 [46.6–59.6] | 36.6 [34.9–38.3] | 47.6 [44.9–50.3] | 30 [29.1–30.9] | 50.9 [47.6–54.1] | 39.2 [38.1–40.3] |
| Mild anemia | - | - | 21.7 [15.2–28.2] | 23.9 [22.3–25.5] | 24.3 [18.7–29.8] | 27 [25.4–28.6] | 25.2 [22.9–27.5] | 27.1 [26.2–28] | 26.7 [23.8–29.6] | 28.3 [27.2–29.3] |
| Severe stunting | 44.1 [33.7–54.5] | 17.5 [16.2–18.8] | 35.4 [28.2–42.7] | 12.2 [11.1–13.3] | 33.3 [27.9–38.8] | 8.5 [7.6–9.4] | 23.4 [21.2–25.5] | 9.3 [8.7–9.8] | 22.8 [20.3–25.3] | 10.9 [10.2–11.5] |
| Moderate stunting | 26.1 [16.9–35.4] | 21.3 [19.9–22.7] | 24.8 [18.3–31.3] | 19.6 [18.2–20.9] | 21.8 [17.1–26.6] | 17 [15.8–18.2] | 21.9 [19.8–24] | 13.1 [12.5–13.8] | 21.4 [18.9–23.8] | 13 [12.3–13.7] |
| Severe underweight | 30.1 [20.5–39.8] | 11.6 [10.5–12.6] | 22.6 [16.3–28.9] | 5.7 [4.9–6.4] | 26.6 [21.5–31.6] | 4.8 [4.1–5.5] | 18.5 [16.6–20.5] | 5.3 [4.9–5.7] | 15.6 [13.5–17.7] | 7.7 [7.1–8.2] |
| Moderate underweight | 37.5 [27.3–47.6] | 18.2 [16.9–19.5] | 42.7 [35.2–50.1] | 17.3 [16-18.6] | 29.6 [24.3–34.8] | 13.5 [12.4–14.6] | 26.6 [24.4–28.8] | 14.4 [13.8–15.1] | 29.1 [26.4–31.7] | 13.4 [12.7–14.1] |
| Severe wasting | 10.4 [4-16.9] | 6.3 [5.5–7.2] | 8.9 [4.6–13.2] | 4.6 [3.9–5.3] | 12.1 [8.3–15.8] | 4.8 [4.1–5.5] | 10.4 [8.8–11.9] | 7.7 [7.2–8.2] | 8.4 [6.7–10] | 8.5 [8-9.1] |
| Moderate wasting | 17.9 [9.9–26] | 12 [10.9–13.1] | 15.8 [10.3–21.3] | 8.4 [7.5–9.4] | 21.7 [16.9–26.4] | 9.5 [8.6–10.4] | 15.4 [13.6–17.3] | 12.1 [11.4–12.7] | 16.3 [14.1–18.5] | 10.1 [9.5–10.7] |

Supplemental table 4: Prevalence (95%) for each outcome by lowest and highest wealth quintile, 1993, 1999, 2006, 2016, and 2021 in rural communities

|  | NFHS 1: 1992–1993 | | NFHS 2: 1998–1999 | | NFHS 3: 2005–2006 | | NFHS 4: 2015–2016 | | NFHS 5: 2019–2021 | |
| --- | --- | --- | --- | --- | --- | --- | --- | --- | --- | --- |
|  | Lowest | Highest | Lowest | Highest | Lowest | Highest | Lowest | Highest | Lowest | Highest |
| ARI | - | - | - | - | 7.2 [6.5-8] | 4.5 [3.4–5.5] | 3.7 [3.5–3.9] | 2.9 [2.5–3.3] | 3.8 [3.6-4] | 3.3 [2.9–3.7] |
| Diarrhea | 11.8 [11-12.7] | 11.8 [10.2–13.3] | 20.4 [19.3–21.5] | 16.6 [14.9–18.4] | 11.9 [11-12.9] | 11.8 [10.1–13.4] | 13.3 [13-13.7] | 11 [10.3–11.7] | 10.9 [10.6–11.3] | 6.8 [6.2–7.4] |
| Severe anemia | - | - | 5.5 [4.7–6.2] | 3.5 [2.4–4.5] | 3.4 [2.8-4] | 2.7 [1.7–3.6] | 2 [1.9–2.2] | 2 [1.7–2.4] | 2.6 [2.4–2.8] | 2.8 [2.4–3.3] |
| Moderate anemia | - | - | 49.4 [47.8–51.1] | 37 [34.3–39.7] | 55.9 [54.2–57.6] | 41.4 [38.4–44.3] | 40.7 [40.1–41.3] | 32.3 [31.1–33.5] | 49.1 [48.5–49.7] | 39.6 [38.3–40.9] |
| Mild anemia | - | - | 23.7 [22.3–25.1] | 24.4 [22-26.7] | 25.6 [24.1–27.1] | 25.6 [23-28.3] | 29.2 [28.7–29.8] | 26.9 [25.8–28] | 27.7 [27.2–28.2] | 28.7 [27.5–29.8] |
| Severe stunting | 39.1 [37.4–40.8] | 13.9 [12-15.8] | 38.9 [37.4–40.4] | 11.9 [10.2–13.5] | 32.2 [30.7–33.6] | 5.3 [4.1–6.5] | 24.6 [24.2–25.1] | 7.2 [6.6–7.8] | 22.2 [21.8–22.7] | 8.7 [8-9.3] |
| Moderate stunting | 21.4 [20-22.9] | 20.1 [17.9–22.3] | 23.6 [22.3–24.9] | 20.3 [18.3–22.4] | 24.1 [22.8–25.4] | 17.4 [15.4–19.5] | 23 [22.6–23.5] | 14 [13.2–14.8] | 20.9 [20.5–21.4] | 12.7 [12-13.5] |
| Severe underweight | 29.6 [28-31.2] | 10 [8.3–11.7] | 28.3 [26.9–29.6] | 5.8 [4.6-7] | 25.5 [24.1–26.9] | 2.8 [1.9–3.7] | 17.7 [17.3–18.1] | 4.6 [4.1–5.1] | 16.1 [15.7–16.5] | 5.1 [4.6–5.7] |
| Moderate underweight | 27 [25.4–28.5] | 18 [15.9–20.1] | 28.9 [27.5–30.2] | 14.3 [12.6–16.1] | 29.8 [28.3–31.2] | 13.3 [11.5–15.2] | 29.5 [29–30] | 13.3 [12.5–14.1] | 25.2 [24.7–25.6] | 11.8 [11.1–12.5] |
| Severe wasting | 10.7 [9.6–11.8] | 6.4 [5-7.7] | 9.8 [8.9–10.7] | 3.5 [2.6–4.4] | 10.7 [9.7–11.6] | 4.1 [3.1–5.2] | 10.6 [10.2–10.9] | 7.1 [6.5–7.6] | 10.3 [9.9–10.6] | 7.1 [6.5–7.7] |
| Moderate wasting | 17.7 [16.4–19.1] | 9.6 [8-11.2] | 17.2 [16-18.3] | 6.9 [5.6–8.2] | 18.7 [17.5–20] | 8.5 [7–10] | 17.6 [17.1–18] | 11.4 [10.7–12.2] | 15.1 [14.7–15.5] | 8.4 [7.8–9.1] |

Supplemental table 5: Prevalence (95%) for each outcome by lowest and highest category of maternal education, 1993, 1999, 2006, 2016, and 2021 in urban communities

|  | NFHS 1: 1992–1993 | | NFHS 2: 1998–1999 | | NFHS 3: 2005–2006 | | NFHS 4: 2015–2016 | | NFHS 5: 2019–2021 | |
| --- | --- | --- | --- | --- | --- | --- | --- | --- | --- | --- |
|  | No schooling | Above 12th grade | No schooling | Above 12th grade | No schooling | Above 12th grade | No schooling | Above 12th grade | No schooling | Above 12th grade |
| ARI | - | - | - | - | 5.9 [5-6.8] | 4 [3.1–4.9] | 2.6 [2.2-3] | 2.2 [1.9–2.5] | 2.4 [1.8-3] | 2.2 [1.8–2.5] |
| Diarrhea | 11.8 [10.7–12.9] | 7.5 [5.9–9.1] | 21.3 [19.5–23] | 12.6 [10.5–14.7] | 12.6 [11.3–13.9] | 9 [7.7–10.4] | 12.9 [12-13.8] | 8.2 [7.5–8.8] | 9 [7.9–10.1] | 5.6 [5.1–6.1] |
| Severe anemia | - | - | 5.9 [4.7–7.1] | 1.4 [0.5–2.3] | 7.3 [6.1–8.6] | 1.6 [0.8–2.3] | 3.5 [2.9-4] | 1.5 [1.2–1.8] | 4.7 [3.8–5.6] | 2.5 [2-2.9] |
| Moderate anemia | - | - | 49.2 [46.7–51.8] | 30.1 [26.7–33.5] | 51.3 [48.9–53.7] | 34.6 [31.8–37.4] | 43.8 [42.3–45.2] | 26.1 [24.9–27.2] | 49.6 [47.4–51.7] | 35.3 [34.1–36.6] |
| Mild anemia | - | - | 22.8 [20.7–24.9] | 22.1 [19.1–25.2] | 22.4 [20.4–24.4] | 27.9 [25.3–30.5] | 26.4 [25.1–27.7] | 26.1 [24.9–27.2] | 24.9 [23.1–26.8] | 28.8 [27.6–30] |
| Severe stunting | 35.1 [33.1–37.1] | 11.9 [9.4–14.4] | 31.2 [29-33.3] | 7.8 [6-9.6] | 25.9 [24-27.8] | 6.5 [5.2–7.8] | 20.3 [19.2–21.4] | 8.1 [7.4–8.8] | 21.4 [19.8–23.1] | 9.7 [9-10.4] |
| Moderate stunting | 21.9 [20.1–23.7] | 16.5 [13.6–19.3] | 23.6 [21.6–25.5] | 14.4 [12.1–16.8] | 22.8 [21-24.6] | 12 [10.3–13.6] | 23.6 [22.4–24.8] | 12.1 [11.3–12.9] | 17.3 [15.8–18.8] | 12.1 [11.3–12.8] |
| Severe underweight | 24 [22.2–25.8] | 7.2 [5.2–9.1] | 19.7 [17.8–21.5] | 4 [2.7–5.3] | 17.9 [16.2–19.6] | 3.6 [2.7–4.6] | 15.6 [14.6–16.6] | 4.3 [3.8–4.8] | 13.2 [11.9–14.5] | 7 [6.4–7.6] |
| Moderate underweight | 27.3 [25.4–29.2] | 12.1 [9.6–14.6] | 28.4 [26.3–30.5] | 12.7 [10.5–14.9] | 26.2 [24.3–28.1] | 9.1 [7.7–10.6] | 25.7 [24.5–27] | 12.1 [11.3–12.9] | 22.6 [21-24.3] | 12.8 [12-13.5] |
| Severe wasting | 9.2 [7.9–10.4] | 4.8 [3.2–6.5] | 6.2 [5.1–7.3] | 4.9 [3.5–6.4] | 9.4 [8.2–10.7] | 4.7 [3.6–5.8] | 9.7 [8.9–10.5] | 8.3 [7.6-9] | 9.4 [8.2–10.5] | 8.2 [7.5–8.8] |
| Moderate wasting | 14.6 [13.1–16.1] | 9.5 [7.2–11.7] | 12.6 [11.1–14.2] | 7 [5.3–8.7] | 15.8 [14.2–17.4] | 7 [5.7–8.3] | 14.9 [13.9–15.9] | 10.9 [10.2–11.7] | 13.3 [11.9–14.6] | 9.8 [9.1–10.5] |

Supplemental table 6: Prevalence (95%) for each outcome by lowest and highest category of maternal education, 1993, 1999, 2006, 2016, and 2021 in rural communities

|  | NFHS 1: 1992–1993 | | NFHS 2: 1998–1999 | | NFHS 3: 2005–2006 | | NFHS 4: 2015–2016 | | NFHS 5: 2019–2021 | |
| --- | --- | --- | --- | --- | --- | --- | --- | --- | --- | --- |
|  | No schooling | Above 12th grade | No schooling | Above 12th grade | No schooling | Above 12th grade | No schooling | Above 12th grade | No schooling | Above 12th grade |
| ARI | - | - | - | - | 7.2 [6.7–7.8] | 3.5 [2-4.9] | 3.4 [3.2–3.6] | 3.4 [3-3.8] | 3.5 [3.2–3.7] | 2.4 [2.1–2.7] |
| Diarrhea | 11.6 [11.1–12.1] | 6.7 [3.6–9.9] | 19.8 [19.1–20.5] | 13.3 [9.7–16.9] | 11.7 [11-12.4] | 12.2 [9.6–14.9] | 12.7 [12.4–13.1] | 12.5 [11.8–13.3] | 10.1 [9.8–10.5] | 7.5 [7–8] |
| Severe anemia | - | - | 5.8 [5.3–6.3] | 2.2 [0.4-4] | 4.2 [3.7–4.7] | 2.5 [0.9-4] | 2.5 [2.3–2.6] | 1.6 [1.2–1.9] | 3.1 [2.9–3.4] | 2.5 [2.2–2.9] |
| Moderate anemia | - | - | 50.4 [49.4–51.5] | 29.8 [24.2–35.3] | 55.5 [54.2–56.7] | 38.7 [33.9–43.6] | 42.5 [41.9–43.1] | 31.6 [30.4–32.8] | 49.9 [49.2–50.6] | 38.7 [37.7–39.7] |
| Mild anemia | - | - | 22.2 [21.3–23.1] | 21.5 [16.5–26.5] | 24.8 [23.8–25.9] | 23.1 [18.9–27.3] | 28.7 [28.2–29.2] | 27.4 [26.2–28.5] | 27.1 [26.4–27.7] | 29.3 [28.4–30.3] |
| Severe stunting | 37 [36–38] | 14.7 [9.5–19.9] | 37.4 [36.4–38.3] | 5.9 [3.3–8.5] | 29.9 [28.9–31] | 3.8 [2.1–5.4] | 24.7 [24.3–25.2] | 8.1 [7.5–8.8] | 23.1 [22.6–23.7] | 10.3 [9.7–10.9] |
| Moderate stunting | 22.4 [21.5–23.2] | 12.1 [7.3–16.9] | 23.7 [22.8–24.5] | 13.9 [10.1–17.7] | 24 [23–25] | 15.7 [12.5–18.9] | 23.2 [22.7–23.7] | 13.9 [13.1–14.7] | 21.3 [20.8–21.9] | 14.4 [13.7–15] |
| Severe underweight | 26.9 [26-27.8] | 6 [2.5–9.5] | 24.9 [24-25.7] | 3.2 [1.3–5.2] | 23.1 [22.1–24.1] | 4.4 [2.6–6.2] | 16.9 [16.5–17.3] | 5 [4.5–5.5] | 16.8 [16.3–17.3] | 6.5 [6-6.9] |
| Moderate underweight | 28.1 [27.2–29] | 10.6 [6.1–15.2] | 27.7 [26.8–28.6] | 11.6 [8.1–15.2] | 28 [27-29.1] | 12.8 [9.9–15.8] | 28.8 [28.3–29.2] | 14.8 [14-15.6] | 24.5 [24-25.1] | 15 [14.4–15.7] |
| Severe wasting | 9.9 [9.3–10.5] | 4.2 [1.2–7.1] | 8.4 [7.9–8.9] | 3.8 [1.7–5.9] | 9.9 [9.3–10.6] | 5 [3.1–6.9] | 9.9 [9.6–10.2] | 7.2 [6.6–7.8] | 10.7 [10.3–11.1] | 8 [7.5–8.5] |
| Moderate wasting | 16.8 [16-17.6] | 5.7 [2.3–9.1] | 14.8 [14.2–15.5] | 8.6 [5.5–11.7] | 17.1 [16.2–18] | 13 [10-15.9] | 16.4 [16-16.8] | 13.5 [12.7–14.3] | 13.9 [13.5–14.4] | 10 [9.5–10.6] |
